# Concurrent pituitary apoplexy and posterior reversible encephalopathy syndrome in a patient with end-stage renal disease on hemodialysis: A case report

**DOI:** 10.1097/MD.0000000000018987

**Published:** 2020-01-31

**Authors:** In Hee Lee, Ho Kyun Kim, Dong Jik Ahn

**Affiliations:** a Department of Internal Medicine; b Department of Radiology, Daegu Catholic University School of Medicine; c Department of Internal Medicine, HANSUNG Union Internal Medicine Clinic and Dialysis Center, Daegu, Republic of Korea.

**Keywords:** end-stage renal disease, hemodialysis, pituitary apoplexy, posterior reversible encephalopathy syndrome

## Abstract

**Rationale:**

Pituitary apoplexy (PA) and posterior reversible encephalopathy syndrome (PRES) are rare neurologic diseases that show acute neuro-ophthalmologic symptoms such as headache, decreased visual acuity, and altered consciousness. These diseases are rarely found in patients with end-stage renal disease (ESRD) on hemodialysis, and simultaneous occurrence of these 2 diseases has not been reported.

**Patient concerns:**

The patient was a 75-year-old man with a history of hypertension, diabetes mellitus, and non-functioning pituitary macroadenoma. He had been receiving hemodialysis for ESRD for 3 months before his presentation to the emergency room. The patient complained of headache, vomiting, and dizziness that started after the previous day's hemodialysis. The patient had voluntarily discontinued his antihypertensive medication 2 weeks before presentation and had high blood pressure with marked fluctuation during hemodialysis. Complete ptosis and ophthalmoplegia on the right side suggested 3rd, 4th, and 6th cranial nerve palsies.

**Diagnoses:**

Magnetic resonance imaging of the brain revealed a pituitary tumor, intratumoral hemorrhage within the sella, and symmetric vasogenic edema in the subcortical white matter in the parieto-occipital lobes. Based on these findings, the patient was diagnosed with PA and PRES.

**Interventions:**

Intravenous administration of hydrocortisone (50 mg every 6 hours after a bolus administration of 100 mg) was initiated. Although surgical decompression was recommended based on the PA score (5/10), the patient declined surgery.

**Outcomes:**

Headache and ocular palsy gradually improved after supportive management. The patient was discharged on the 14th day of hospitalization with no recurrence 5 months post-presentation. Current therapy includes antihypertensive agents, oral prednisolone (7.5 mg/day), and maintenance hemodialysis.

**Lessons:**

Neurologic abnormalities developed in a patient with ESRD on hemodialysis, suggesting the importance of prompt diagnosis and treatment in similar instances.

## 1 Introduction

Pituitary apoplexy (PA) is a rare clinical syndrome characterized by severe headache, visual impairment, ocular paresis, and an altered mental state due to hemorrhage or infarction in the pituitary gland.^[1]^ PA is mostly found in patients with an underlying pituitary tumor and has various clinical manifestations ranging from a benign course to permanent neuropathy or mortality.^[1]^ Posterior reversible encephalopathy syndrome (PRES) is a syndrome characterized by vasogenic edema on neuroimaging along with acute or subacute confusion, sudden headache, visual disturbances, and seizure. PA cases^[2,3]^ or PRES cases^[4–6]^ have been rarely reported in patients with end-stage renal disease (ESRD) undergoing dialysis therapy. Moreover, simultaneous occurrence of PA and PRES in ESRD patients has not been reported. Here, we report a case of PA accompanied by PRES in a 75-year-old man with ESRD on hemodialysis.

## 2 Case report

**Figure 1:**
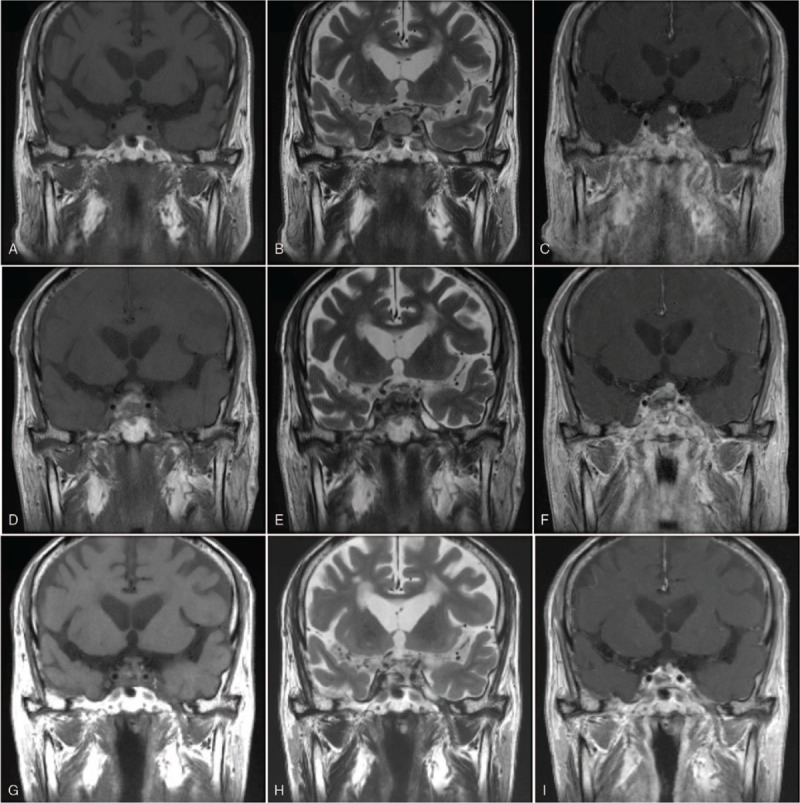
(A–C) Contrast enhanced coronal T1-WI of the initial brain MR imaging show just eccentric focal enhancement in the inferior aspect of the pituitary tumor. (D–F) Five days later, the pituitary mass shows complex signal intensities with hyperintensity on T1-WI and hypointensity on T2-WI, representing intratumoral hemorrhage. (G–I) In a follow-up brain MR imaging obtained at 5 months after discharge, hemorrhagic lesions within the sella were resolved markedly. MR = magnetic resonance, WI = weighted images.

**Figure 2:**
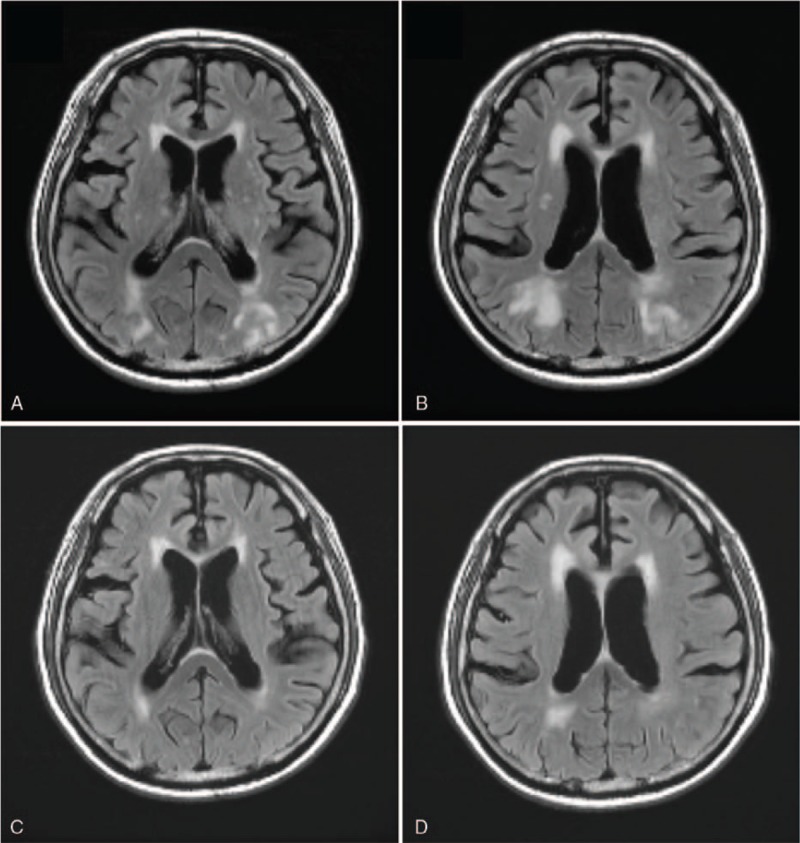
(A, B) FLAIR sequences of the brain magnetic resonance (MR) imaging at presentation show bilateral T2 hyperintensities in the cortex and subcortical white matters of the parieto-occipital lobes and along the watershed zones, suggestive of extensive vasogenic edema. (C, D) FLAIR images of brain MRI performed 5 months after discharge represent a significant regression of previous cerebral lesions. FLAIR = Fluid-attenuated inversion recovery.

A 75-year-old man visited the emergency room of our hospital with chief complaints of severe headache, vomiting, and dizziness that started immediately after hemodialysis the previous day. The patient had been receiving oral hypoglycemic agents and antihypertensive agents (telmisartan 80 mg/day and bisoprolol 10 mg/day) since being diagnosed with diabetes mellitus and hypertension 20 years earlier. Although the patient had been diagnosed with a non-functioning pituitary macroadenoma 2 years earlier, there were no specific symptoms, and no specific medications had been prescribed. The patient had received regular hemodialysis with systemic heparinization (4 hours/session, 3 sessions/week) for ESRD secondary to diabetic nephropathy that had developed 3 months before emergency room presentation. The usual range of interdialytic weight gain was 1.5 to 2.0 kg. The pre- and post-dialysis blood pressure (BP) measurements were 200/110 mmHg (mean BP, 143 mmHg) and 120/70 mmHg (mean BP, 87 mmHg), respectively, showing a marked fluctuation in BP during the previous hemodialysis. The patient had refused antihypertensive therapy during the 2 weeks before hospital presentation. At the time of admission, the vital signs were a BP of 110/70 mmHg, pulse rate of 101/minute, respiration rate of 20/minute, and body temperature of 35.8°C. The patient was lethargic (Glasgow coma scale, 12/15) and complained of severe headache, particularly in the bi-frontal area and right retro-orbital region (numeric pain rating scale, 7/10). Neurologic examination revealed a pupillary size of 3 mm/4 mm with normal light reflexes; complete ptosis and ophthalmoplegia were present on the right side. There were no occurrences of neck stiffness, focal neurologic deficits, or seizure. A peripheral blood test at admission showed the following: white blood cell count, 6,300/μL (neutrophils 59%); hemoglobin, 10.5 g/dL; platelet count, 142,000/μL; and an erythrocyte sedimentation rate of 20 mm/hour. Serum biochemical examination revealed the following: glucose, 126 mg/dL; blood urea nitrogen, 45.8 mg/dL; creatinine, 6.6 mg/dL; total protein, 5.2 g/dL; albumin, 3.1 g/dL; total cholesterol, 154 mg/dL; aspartate aminotransferase, 18 IU/L; alanine aminotransferase, 12 IU/L; calcium, 8.2 mg/dL; phosphorus, 4.3 mg/dL; uric acid, 5.4 mg/dL; Na^+^/K^+^/Cl^−^, 136/4.8/95 mEq/L; magnesium, 2.2 mg/dL; C-reactive protein, 3 mg/L; and a HbA_1_C level of 6.5%. Serologic test results for HBs Ag, anti-HCV Ab, anti-HIV Ab, anti-nuclear Ab, ds-DNA Ab, and Venereal Disease Research Laboratory (VDRL) test were all negative. Results from serologic tests were all negative. Since the clinical signs suggested central nervous system (CNS) disease, we performed brain computed tomography (CT) and brain magnetic resonance (MR) imaging. Initial brain MR imaging revealed a large pituitary tumor of 2.0 × 1.4 cm with internal hemorrhage within the sella, suggestive of PA (Fig. 1A–C). In addition, we identified multiple areas of patchy-type vasogenic edema at the subcortical and deep white matter of the bilateral parieto-occipital lobes (Fig. 2A, B). On the basis of these findings, the patient was diagnosed with simultaneous occurrence of PA and PRES, and we immediately performed serum hormone tests. The serum hormone test results at the time of admission and 3 months earlier are described in Table 1. In addition, supportive management was initiated. The patient was treated with intravenous (IV) 50 mg hydrocortisone every 6 hours (200 mg/day) after IV administration of a 100 mg bolus of hydrocortisone. This was followed by gradual tapering to 100 mg/day and then 50 mg/day at 2-day intervals. On the 2nd day after admission to the division of nephrology, headache, nausea, vomiting, and dizziness persisted. Neurologic examination results led to diagnosis of palsies of the 3rd, 4th, and 6th cranial nerves with a PA score of 5/10.^[1,7]^ Surgical decompression was recommended, but the patient refused surgery because of his old age and poor systemic condition. On the 5th hospital day, a follow-up brain MR imaging was performed (Fig. 1D–F). After conservative treatment, including IV administration of corticosteroids, analgesics, and antihypertensive agents, nausea and vomiting were mitigated, and the headache lessened. His consciousness also improved. Because the patient became clinically stable on the 8th day of hospitalization, the IV corticosteroid administration ceased, and the patient was maintained on oral prednisolone (10 mg/day). During the hospitalization, the patient's hemodialysis treatment was maintained, and BP was controlled to below 140/90 mmHg by addition of an antihypertensive agent (nifedipine 66 mg/day). Neurological complications, such as headache and ocular palsy, as well as systemic symptoms improved, and the patient was discharged on the 14th hospital day. In a follow-up brain MR imaging obtained at 5 months after discharge, lesions of PA and PRES were found to have markedly improved, respectively (Figs. 1G–I and 2C, D). The patient is currently receiving antihypertensive medications, oral prednisolone (7.5 mg/day), and maintenance hemodialysis with no significant signs of neurologic sequelae.

**Table 1:**
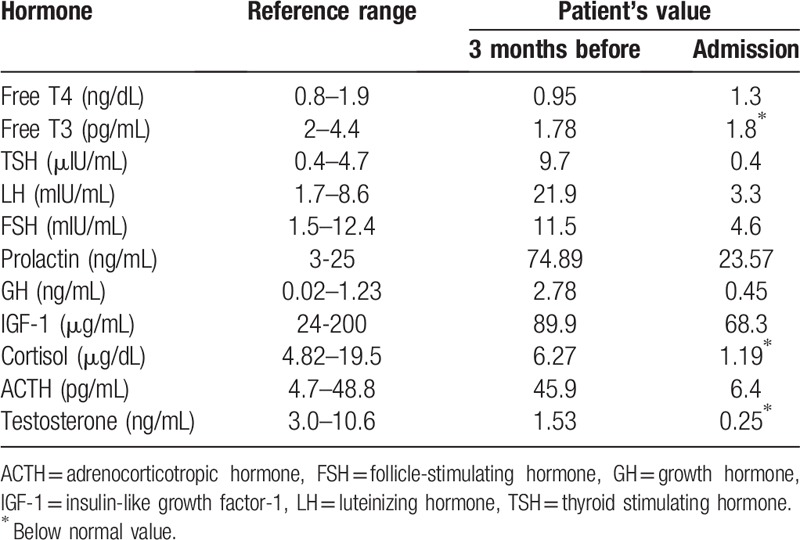
Serum hormone levels of the patient.

## 3 Discussion

PA is a disease in which the pituitary gland rapidly expands due to pituitary hemorrhage, infarction, and necrosis, causing compression of the proximal regions of suprasellar structures or signs of meningeal irritation.^[1]^ PA occurs in 1.6% to 2.8% of patients with pituitary macroadenoma, with a male to female ratio of 2:1, and affects individuals in their 50s to 60s.^[8]^ Factors that predispose to PA, most commonly severe hypertension, are observed in 40% of cases. Other predisposing factors, including major surgery and anticoagulant administration, are also observed.^[7]^ The predisposing factors of the patient in this case were uncontrolled hypertension, diabetes mellitus, chronic kidney disease, and pituitary macroadenoma.^[1,9]^ Liu et al analyzed 288 patients with pituitary adenoma and found pituitary hemorrhage in 81 (28%). ESRD was also reported to be a significant risk factor for pituitary hemorrhage along with macroadenoma (>10 mm), non-functioning adenoma, antihypertensive therapy, and dopamine agonist administration.^[10]^ Since sudden fluctuation of BP and use of anticoagulants, such as heparin, during hemodialysis are known major risk factors for pituitary hemorrhage or PA,^[3,11]^ we hypothesized that the PA in this patient may have been primarily due to an underlying pituitary adenoma and dialysis therapy. The most common symptom of PA is severe headache in the retroorbital and bifrontal regions accompanied by nausea and vomiting.^[7,12]^ In addition, as in the present case, optic abnormalities and multiple cranial nerve palsies are also common signs of PA.^[7,13]^ Once an acute neurologic disorder is identified in patients with an underlying pituitary tumor, rapid performance of radiologic examinations such as brain CT and brain MR imaging is imperative to assess presence of pituitary hemorrhage and rule out more common CNS disorders.^[7]^ Higher incidences of dialysis-related neurologic complications accompany severe hemodynamic changes during hemodialysis and are associated with increased morbidity and mortality in these patients.^[14,15]^ Like our patient, in case of PA accompanied by intratumoral hemorrhage, different intensities of brain MR imaging can be observed depending on the process of hemorrhagic degeneration. Within the first 7 days of PA, isointense or slightly hypointense signal in T1-weighted images (WI) and hypointense lesions in T2-WI are depicted. In the subacute stage, from 1 to 3 weeks, hyperintensity due to methemoglobin is visualized in T1-WI and T2-WI. After 3 weeks, strong hypointensity in both T1-WI and T2-WI, secondary to the accumulation of hemosiderin and ferritin, may be noted.^[8]^

In most cases of PA, necrosis occurs in the anterior lobe of the pituitary gland and causes hormone deficiencies, particularly decreased prolactin production. Hormone deficiencies suggest a poor prognosis for complete recovery of pituitary function.^[14,16]^ Our patient's pituitary hormone levels, with the exception of decreased serum cortisol, were within normal limits at the time of presentation (Table 1). Because of the severe implications of acute secondary adrenal insufficiency, a supraphysiologic dose of a corticosteroid is indicated during the acute phase of PA.^[7]^ Corticosteroid administration is also indicated when signs of hemodynamic instability, altered consciousness, decreased visual acuity, and severe defects in the visual field are present.^[7,17]^ Supportive management in addition to hormone replacement is recommended in the acute stage of PA. Elective surgery should be considered in cases of no clinical improvement, progression of visual field defects or vision loss, and aggravation of neurologic signs after 1 week of corticosteroid administration. Neurosurgical decompression has been reported to yield a favorable clinical outcome.^[7]^ We recommended surgical decompression on the 2nd day of hospitalization because the patient's PA score was 5 points.^[1,7]^ However, the patient refused surgery. Fortunately, conservative therapy gradually alleviated the vomiting, headache, and progression of neuro-ophthalmic complications; and monitoring for signs and symptoms of relapse ensued.^[18]^

PRES is a disorder in which vasogenic edema in the cerebral white matter causes seizures, headache, nausea, vomiting, visual disturbances, altered consciousness, and focal neurologic deficits.^[19]^ PRES has a number of causes that, on rare occasions, includes chronic kidney disease or acute kidney injury that results in uremia, intravascular volume expansion, electrolyte disorders, and hypertensive crisis.^[4–6,20]^ On brain MR imaging, PRES presents as cortical and/or subcortical lesions with iso-intense or low signal intensity in T1-WI and hyperintense signal in T2-WI and FLAIR sequences.^[19]^ In most cases of PRES, vasogenic edema gradually subsides after correction of risk factors and treatment of underlying disease.^[21]^ However, ESRD patients on dialysis are more likely to develop permanent neurologic sequelae or mortality due to increased vulnerability to microvascular brain injury.^[5]^ Thus, active treatment for risk factors, including hypertension, is required after prompt diagnosis. Considering this patient's clinical presentations and the evidence of radiologic resolution, all diagnostic criteria of PRES were satisfied.^[21]^ PA was diagnosed as the cause of this patient's ophthalmologic disorders, cranial nerve deficits, and decreased serum cortisol level. Marked variability of BP with systemic heparinization during hemodialysis is associated with PRES as well as PA. We surmised that the conservative treatment for PA of this patient also contributed to the improvement of PRES. However, the absence of previous reports on the pathogenic association between PA and PRES prevents the conclusion of their coincidence in this patient.

In summary, a 75-year-old male ESRD patient with an underlying pituitary macroadenoma was diagnosed with both PA and PRES during hemodialysis; these conditions improved with supportive therapy. This case suggests that ESRD patients on hemodialysis may be more susceptible to various neurologic diseases due to severe hypertension, systemic heparinization, and hemodynamic instability. Thus, this case demonstrates the importance of prompt diagnosis and treatment for ESRD with dialysis-related neurologic complications.

## Author contributions

**Conceptualization:** In Hee Lee.

**Data curation:** Ho Kyun Kim, Dong Jik Ahn.

**Formal analysis:** In Hee Lee.

**Methodology:** In Hee Lee.

**Validation:** Ho Kyun Kim, Dong Jik Ahn.

**Writing – original draft:** In Hee Lee.

**Writing – review & editing:** In Hee Lee.

In Hee Lee orcid: 0000-0003-3562-7586.

## References

[1] Glezer A Bronstein MD Pituitary apoplexy: pathophysiology, diagnosis and management. Arch Endocrinol Metab 2015;59:259–64. 26154095 10.1590/2359-3997000000047

[2] Mohr G Hardy J Hemorrhage, necrosis and apoplexy in pituitary adenomas. Surg Neurol 1982;18:181–9. 7179072 10.1016/0090-3019(82)90388-3

[3] De la Torre M Alcázar R Aquirre M The dialysis patient with headache and sudden hypotension: consider pituitary apoplexy. Nephrol Dial Tranplant 1998;13:787–8. 10.1093/ndt/13.3.787 9550672

[4] Gokce M Dogan E Nacitarhan S Posterior reversible encephalopathy syndrome caused by hypertensive encephalopathy and acute uremia. Neurtocrit Care 2006;4:133–6. 10.1385/NCC:4:2:133 16627901

[5] Canney M Kelly D Clarkson M Posterior reversible encephalopathy syndrome in end-stage kidney disease: not strictly posterior or reversible. Am J Nephrol 2015;41:177–82. 25871433 10.1159/000381316

[6] Aatif T El Farouki MR Benyahia M Posteroir reversible encephalopathy syndrome in a hypertensive patient with renal failure. Saudi J Kidney Dis Transpl 2016;27:411–4. 26997402 10.4103/1319-2442.178586

[7] Rajasekaran S Vanderpump M Baldeweg S UK guidelines for the management of pituitary apoplexy. Clin Endocrinol 2011;74:9–20. 10.1111/j.1365-2265.2010.03913.x 21044119

[8] Wildemberg LE Glezer A Bronstein MD Apoplexy in nonfunctioning pituitary adenomas. Pituitary 2018;21:138–44. 29383476 10.1007/s11102-018-0870-x

[9] Biousse V Newman NJ Oyesiku NM Precipitating factors in pituitary apoplexy. J Neurol Neurosurg Psychiatry 2001;71:542–5. 11561045 10.1136/jnnp.71.4.542 PMC1763528

[10] Liu ZH Tu PH Pai PC Predisposing factors of pituitary hemorrhage. Eur J Neurol 2012;19:733–8. 22182406 10.1111/j.1468-1331.2011.03619.x

[11] Lin CM Lin JW Tsai JT Intracranial pressure fluctuation during hemodialysis in renal failure patients with intracranial hemorrhage. Acta Neurochir Suppl 2008;101:141–4. 18642649 10.1007/978-3-211-78205-7_24

[12] Ayuk J McGregor EJ Mitchell RD Acute management of pituitary apoplexy-surgery or conservative management? Clin Endocrinol 2004;61:747–52. 10.1111/j.1365-2265.2004.02162.x 15579190

[13] Sibal L Ball SG Connolly V Pituitary apoplexy: a review of clinical presentation, management and outcome in 45 cases. Pituitary 2004;7:157–63. 16010459 10.1007/s11102-005-1050-3

[14] Rizzo MA Frediani F Granata A Neurologic complications of hemodialysis: state of the art. J Nephrol 2012;25:170–82. 22241633 10.5301/jn.5000087

[15] Reeves PB Mc Causland FR Mechanisms, clinical implications, and treatment of intradialytic hypotension. Clin J Am Soc Nephrol 2018;13:1297–303. 29483138 10.2215/CJN.12141017 PMC6086712

[16] Zayour DH Selman WR Arafah BM Extreme elevation of intrasellar pressure in patients with pituitary tumor apoplexy: relation to pituitary function. J Clin Endocrinol Metab 2004;89:5649–54. 15531524 10.1210/jc.2004-0884

[17] Briet C Salenave S Bonneville JF Pituitary apoplexy. Endocr Rev 2015;36:622–45. 26414232 10.1210/er.2015-1042

[18] Capatina C Inder W Karavitaki N Management of endocrine disease: pituitary tumour apoplexy. Eur J Endocrinol 2015;172:R179–90. 25452466 10.1530/EJE-14-0794

[19] Grag RK Posterior leukoencephalopathy syndrome. Postgrad Med J 2001;77:24–8. 11123390 10.1136/pmj.77.903.24 PMC1741870

[20] Fugate JE Claassen DO Cloft HJ Posterior reversible encephalopathy syndrome: associated clinical and radiologic findings. Mayo Clin Proc 2010;85:427–32. 20435835 10.4065/mcp.2009.0590 PMC2861971

[21] Fischer M Schmutzhard E Posterior reversible encephalopathy syndrome. J Neurol 2017;264:1608–16. 28054130 10.1007/s00415-016-8377-8 PMC5533845

